# Effect of cold atmospheric plasma (CAP) on human adenoviruses is adenovirus type-dependent

**DOI:** 10.1371/journal.pone.0202352

**Published:** 2018-10-26

**Authors:** Oskar Bunz, Kemal Mese, Wenli Zhang, Andree Piwowarczyk, Anja Ehrhardt

**Affiliations:** 1 Institute of Immunology, Centre for Biomedical Education and Research (ZBAF), School of Medicine, Faculty of Health, Witten/Herdecke University, Witten, Germany; 2 Department of Prosthodontics, School of Dentistry, Faculty of Health, Witten/Herdecke University, Witten, Germany; 3 Institute of Virology and Microbiology, Centre for Biomedical Education and Research (ZBAF), School of Human Medicine, Faculty of Health, Witten/Herdecke University, Witten, Germany; Massachusetts General Hospital, UNITED STATES

## Abstract

More than 70 human adenovirus types were identified divided into 7 different species (A-G). Diseases caused by human adenoviruses are type-dependent and can range from mild to severe respiratory infections, gastrointestinal infections or eye infections such as epidemic keratoconjunctivitis. Unfortunately there is no specific anti-adenovirus therapy available. Here we addressed the question whether treatment with cold atmospheric plasma (CAP) for anti-adenoviral therapy such as virus-mediated ulcerations may be feasible. CAP has already been explored for the treatment of dermatological diseases such as chronic wounds. To investigate whether CAP is an effective antiviral tool, purified human adenovirus types derived from different human adenovirus species (HAdV -4, -5, -20, -35, -37, -50) tagged with luciferase were treated with defined dosages of plasma. The CAP treatment was varied by incrementally increasing the time span of CAP treatment. After CAP treatment, the virus containing solution was added to eukaryotic cells and the viral load was determined by measurement of luciferase expression levels. Through the plasma treatment the adenovirus driven luciferase expression directly correlating with adenovirus transduction efficiencies could be reduced for HAdV-5 and HAdV-37. Plasma treatment had no influence on adenovirus derived luciferase expression levels for HAdV-4 and HAdV-50 and it even had a positive effect on luciferase expression levels for HAdV-20 and HAdV-35. These results suggest that CAP has a type dependent effect on adenoviruses and that infectivity can be even increased for certain adenovirus types. Further studies should address the mechanisms behind this phenomenon. In summary we demonstrate that CAP may represent an interesting option for antiviral treatment in a virus type dependent manner.

## Introduction

Plasma is the fourth state of matter and has properties unlike those of the other states (solid, liquid, gas), which is attributed to the partial or complete ionization of the gas. The emission by plasma consists of radiation such as visible light, UV, electromagnetic waves. Furthermore it contains ions and electrons, free radicals and other reactive species like reactive oxygen and nitrogen species (RONS). In reaction with water those reactive species can induces hydrogen peroxide.

The production of cold atmospheric plasma (CAP) or more precisely non-thermal atmospheric-pressure plasma is feasible since the 1990s, based on oxygen, argon, helium and even pure air plasmas are in use. In contrast to “hot plasma”, which is produced through high temperatures and which is characterized by fully ionized gas, CAP is only partially ionized gas and temperatures are below 40°C making it suitable for medical purposes. An increasing body of evidence revealed that CAP might be suitable for the treatment of chronic wounds [1]. On the one hand, CAP has a broad bacteriostatic and bactericidal effect due to emission of UV light and generation of reactive oxygen/ nitrogen species thus reducing the bacterial load in the wound. Conjointly, CAP has been demonstrated to induce proliferation of a basal epidermal keratinocyte cell line [2], which in turn will foster the wound healing process. In addition to this, CAP has already successfully used in the treatment of dermatological diseases, like acne vulgaris [3] and in vitro studies demonstrated that CAP could induce apoptosis in tumor cells [4].

Because of its bacteriostatic and bactericidal effects CAP might be a suitable technique in medical and dental practice, e.g., for the treatment of disorders being associated with acute or chronic inflammation. With respect to antiviral effects of CAP attention seems to be on the treatment of food to prevent transmissible viral diseases. For instance, in an in vitro study the effect of the argon gas plasma jet on norovirus was investigated [5]. Moreover, in an ex vivo study, norovirus positive human stool samples were treated by barrier discharge plasma, whereby a slight reduction in the viral load could be detected [6]. Besides norovirus also herpesviruses were studied in vitro and a reduction of herpes simplex virus type 1 on corneal epithelial cells could be detected [7]. In addition a randomized placebo-controlled clinical trial showed that CAP has a slight effect on pain and improves healing in the first days for patients suffering on herpes zoster [8].

Here we tested whether CAP treatment may be suitable as an antiviral agent and exemplified this idea for a range of different human adenovirus types. Adenoviruses comprise several hundred agents infecting reptiles, fishes, birds and mammals. There are more than 70 types of human adenoviruses (HAdV) which can be divided into 7 genetically highly diverse species (A to G). Human Adenoviruses (HAdVs) are common pathogens causing a multitude of disease manifestations in a type-dependent manner, which are usually not fatal and self-limiting in immune-competent patients. They are widespread pathogens of the upper and lower respiratory tracts, the digestive and urinary tracts and affecting internal organs and exacerbate asthmatic conditions and chronic obstructive pulmonary disease, cause highly infectious follicular epidemic keratoconjunctivitis or fatal disease in immune-compromised patients [9]. No effective treatment is available against HAdV infections and therefore novel treatment options are urgently needed. In the present study different HAdVs derived from different species were treated with plasma-treated and transduction efficiencies were measured by reporter assays. To our surprise we observed that the effect of plasma on HAdVs was type dependent and that plasma may only represent a treatment option for specific adenovirus types.

## Materials and methods

### Cell culture

The Chinese Hamster Ovary cells (CHO cells) were cultured under a humidified atmosphere of 5% CO_2_ and 37°C in tissue culture dish until confluency was reached. The nutrient medium consisted in DMEM medium (PAN-Biotech GmbH, Aidenbach, Germany) with 10% FCS (PAN-Biotech GmbH, Aidenbach, Germany) and 1% Penicillin/Streptomycin (PAN-Biotech GmbH, Aidenbach, Germany). CHO cells were counted and seeded at a density of 3x10^4^ per well in 96-well tissue culture plates in triplicates for each test group.

### Virus production

Reporter tagged HAdVs were described in our previous publication [10]. In brief, recombinant HAdVs based on different types containing a luciferase encoding transgene in the E3 region were amplified in HEK293 cells cultured in DMEM medium (PAN-Biotech GmbH, Aidenbach, Germany) with 10% FCS (PAN-Biotech GmbH, Aidenbach, Germany) and 1% Penicillin/Streptomycin (PAN-Biotech GmbH, Aidenbach, Germany). Crude lysates were used to purify virions using cesium chloride (CsCl) gradient-based ultracentrifugation (Beckman Coulter), which was followed by a desalting step based on disposable PD-10 desalting columns (GE Healthcare, Germany). Purified viruses were stored in aliquots at -80°C for further use. Adenovirus particle concentrations were determined by measuring the optical density at 260 nm and expressed as viral particles (vps) per microliter as described previously [11].

### Preparation of the virus suspension

Based on the measured adenovirus particle concentrations, we performed preliminary experiments to determine an individual virus particle number per cell with the highest possible luciferase expression which was set for each used type of adenovirus. Serial 10-fold dilutions of each virus type were prepared and luciferase expression levels measured.

### Plasma treatment

**Fig 1:**
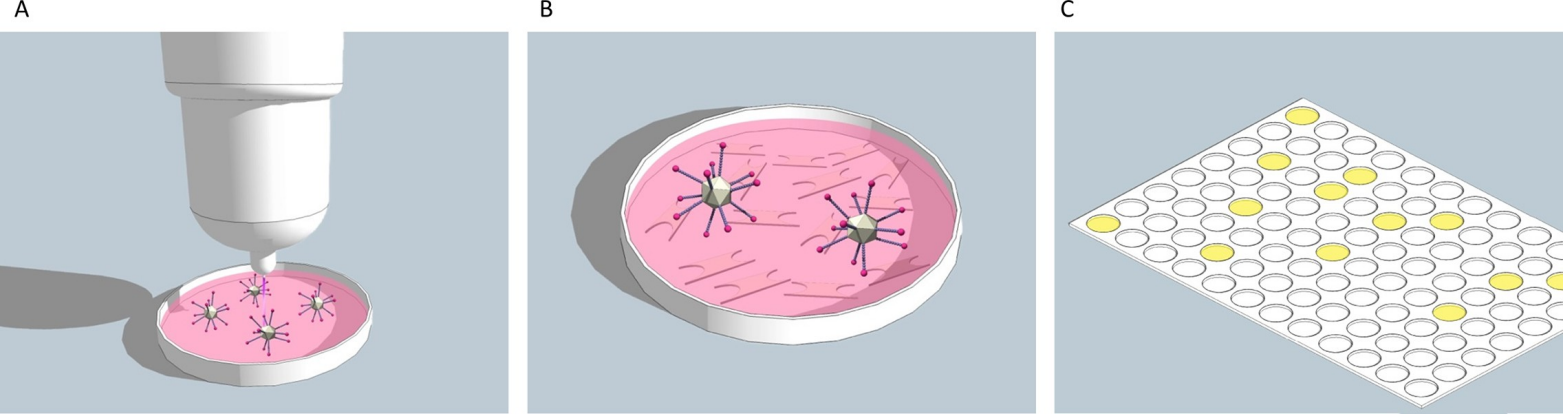
Outline of the experimental setup. **(A)** The virus-medium suspension was treated with cold atmospheric plasma (CAP). **(B)** CAP treated virus suspension was pipetted onto the CHO cells. **(C)** After 26 h incubation a luciferase assay was performed.

The treatment of the adenoviruses with cold atmospheric plasma (kINPen med, neoplas GmbH, Greifswald, Germany) was carried out by treating a virus-medium suspension. 500 μl medium per well (24 well-plate) was treated by different time doses (no treatment, 30 sec., 60 sec., 90 sec., 120 sec., 150 sec.). A distance of 15 mm to the medium surface was maintained (Fig 1A). The CAP engine was operated with argon gas (purity 4.8) with the entry pressure set to 2.5 bar and the flow rate set to 5 L/min.

### Infection of CHO cells

100 μl of the CAP treated virus suspension were directly pipetted onto the CHO cells per well (96-well format) (Fig 1B). After infection, the cells were cultured 26 h under a humidified atmosphere of 5% CO^2^ and 37°C.

### Evaluation of adenovirus transduction efficiency after CAP treatment

The transduction efficiencies of tagged adenoviruses in CHO cells after treatment with CAP were measured by determining the reporter gene (luciferase) expression levels. 26 h after infection luciferase activity was measured (Fig 1C) with the Nano-Glo assay system (Promega GmbH, Mannheim, Germany). The luminescence was detected quantified using a respective plate reader (Genios, Tecan Group Ltd, Männedorf, Switzerland).

### Measurement of pH values after CAP treatment

The medium was stored at room temperature. 500 μl medium per well (24 well-plate) was CAP-treated by different time doses (30, 60, 90, 120, and 150 seconds) without adding virus. A distance of 15 mm to the medium surface was maintained. Immediately after treatment samples were kept at 37°C and 5% CO_2_ during the time course of the experiment. As a control untreated medium was used. After calibration, pH values were measured by pH-Meter (inoLab pH 720, Xylem Analytics Germany Sales GmbH & Co. KG, WTW, Weilheim, Germany). pH values were measured 5, 15, 30, 60, 120, and 180 minutes and 24 hours after treatment.

### Temperature measurement after CAP treatment

The medium was stored at room temperature and treated with CAP for 30, 60, 90, 120 and 150 seconds. A digital temperature measurement was performed 30, 60, 90, 120 and 150 seconds post-treatment.

### Statistical analysis

Each experiment was repeated three times. From the triplicates of each test group, a mean value was formed by Excel. Subsequently, these mean values could be related to the untreated positive control and the three independent experiments could be presented in one graph with error indicators. All data are reported as mean +/- SEM (standard error of the mean). Statistical comparison was performed using the two-tailed student’s test and a value of *p* < 0.05 was considered to be relevant compared to the respective control group.

## Results

### Experimental setup and characterization of used adenoviral vectors

To test whether plasma treatment has an effect on human adenoviruses we elected 6 HAdVs derived from different adenovirus species: HAdV-5 from species C, HAdV-35 and HAdV-50 from species B, HAdV-20 and HAdV-37 from species D, and HAdV-4 from species E. After amplification and purification, viral particle numbers (vps) per microliter of each HAdV were determined and are listed in **Table 1**. Initially we optimized the used viral dose for infection experiments to assure that after infection of CHO cells with reporter tagged viruses luciferase expression levels were in the non-saturated range (data not shown). Based on these results applied vps per cell for each virus were determined. As summarized in **Table 1** an up to 4-fold difference of the used virus dose was applied.

**Table 1:** Titers of applied viruses and used virus dose for experiments.

Adenovirus type	vps/μl	Applied vps/cell
HAdV-4	1,4E+09	500
HAdV-5	3,79E+08	1000
HAdV-20	1,08E+09	200
HAdV-35	8,1E+08	2000
HAdV-37	1,51E+09	500
HAdV-50	1,32E+08	2000

Viral particles (vps) measured by optical density at 260 nm and the optimized vps number per cell applied in experiments are provided.

### CAP treatment of HAdV is adenovirus type-dependent

**Fig 2:**
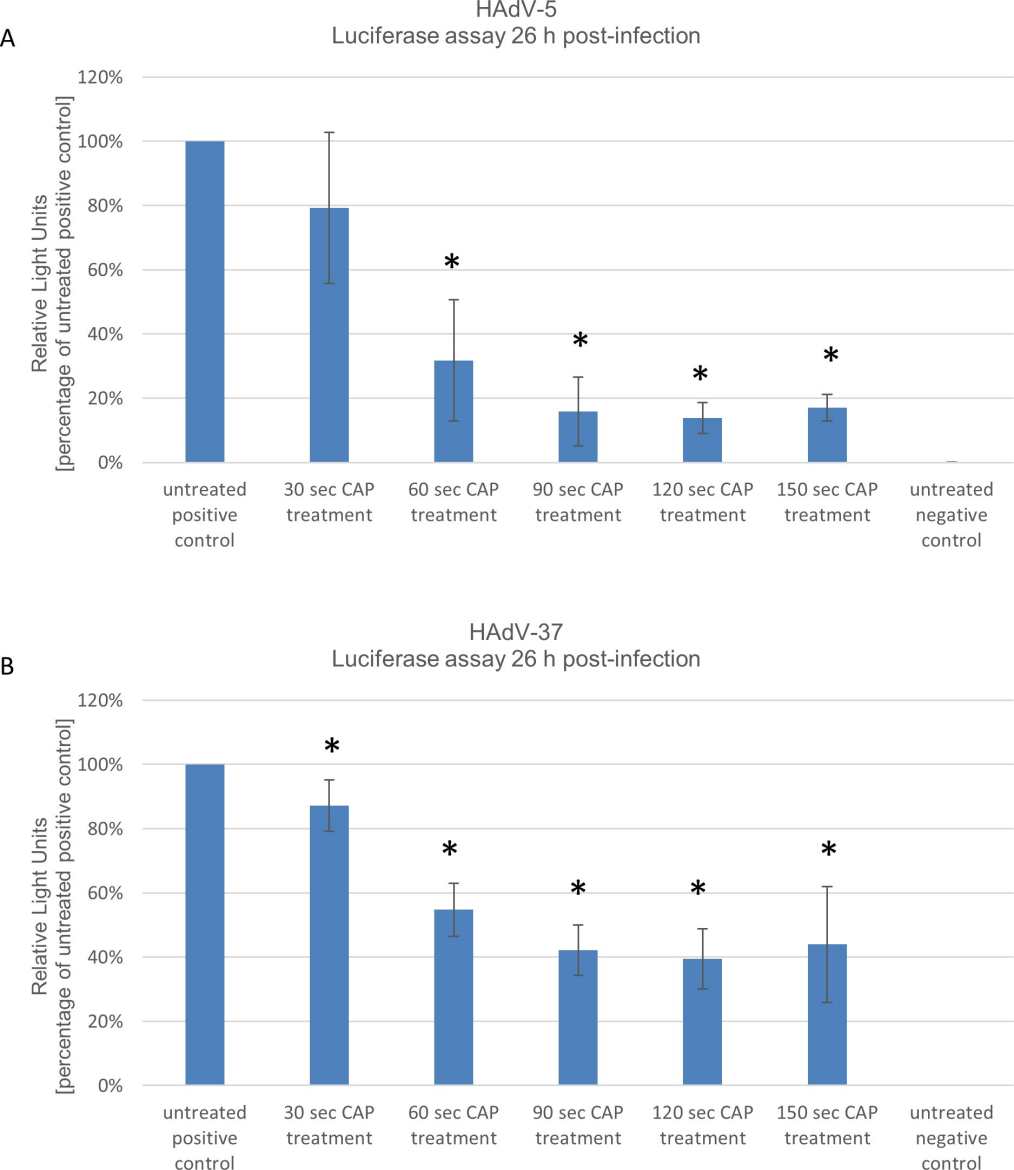
Human adenovirus (HAdV) types HAdV-5 and HAdV-37 show decreased transduction efficiencies after treatment with cold atmospheric plasma (CAP). Viruses were treated with CAP for the given times and CHO cells were infected. 26 hrs post-infection luciferase values were measured. **(A)** HAdV-5 treated with CAP and **(B)** HAdV-37 treated with CAP. Positive control: untreated virus; negative control: no virus. Mean +/- standard deviation are shown. * P-value of <0.05 was considered to be significant. Experiments were performed in triplicates and repeated 3 times.

First we evaluated the commonly studied HAdV-5. CHO cells were infected at 1000 vps/cell and luciferase levels were measured 26 hrs post-infection. As shown in **Fig 2A** virus transduction efficiencies expressed as luciferase expression levels can be reduced to up to 14% of original levels after CAP treatment for 120 seconds. Also for HAdV-37 driven luciferase expression using the identical experimental setup, luciferase levels could be reduced to up to 40% after CAP treatment for 120 seconds compared to the positive control (**Fig 2B**).

**Fig 3:**
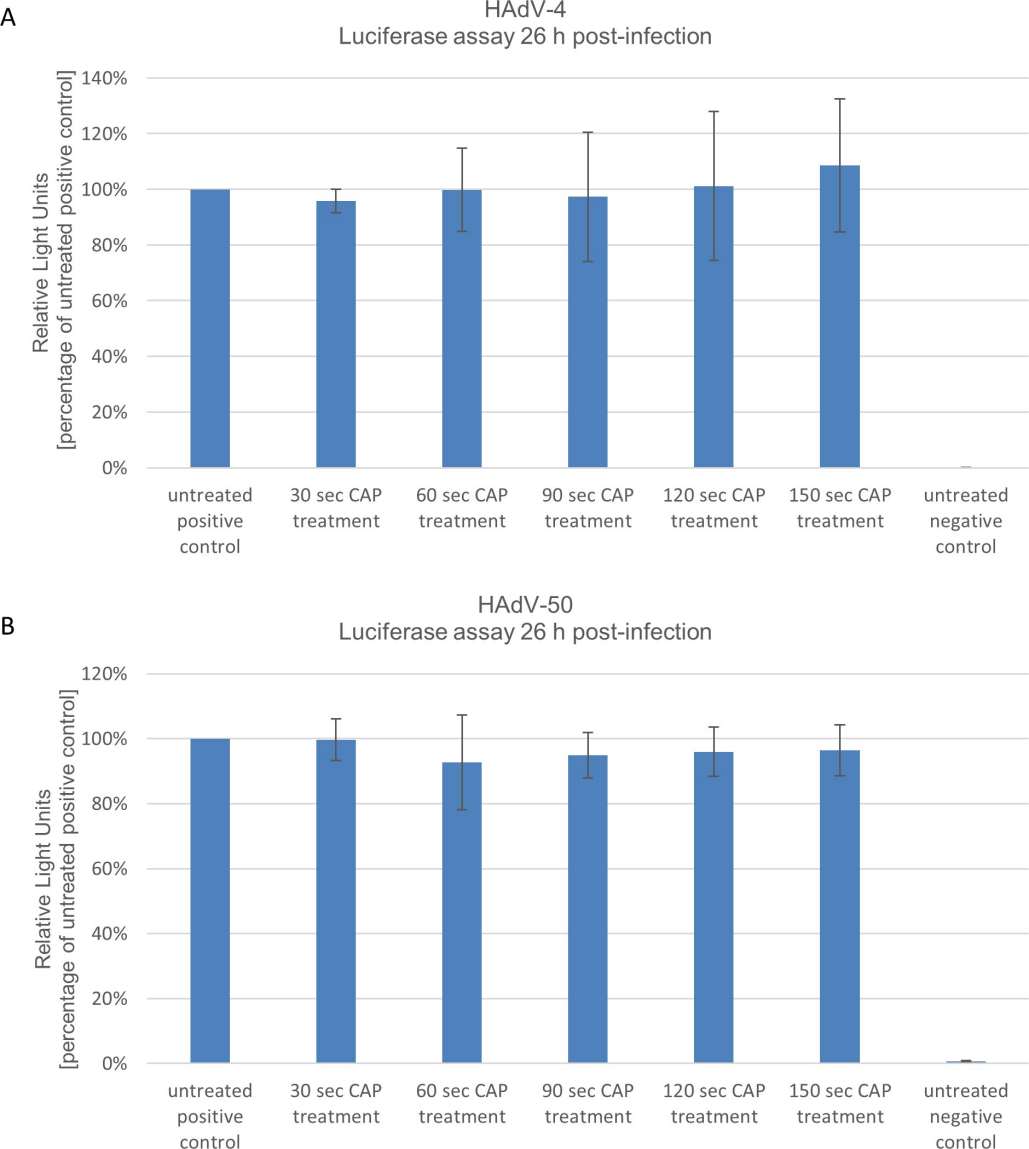
Human adenovirus (HAdV) types HAdV-4 and HAdV-50 revealed unchanged transduction efficiencies after could atmospheric plasma (CAP) treatment. CHO cells were infected with plasma treated viruses. **(A)** Results for CAP treated HAdV4. **(B)** Relative luciferase expression levels for CAP treated HAdV-50. Positive control: untreated virus; negative control: no virus. Mean +/- standard deviation are shown. Experiments were performed in triplicates and repeated 3 times.

**Fig 4:**
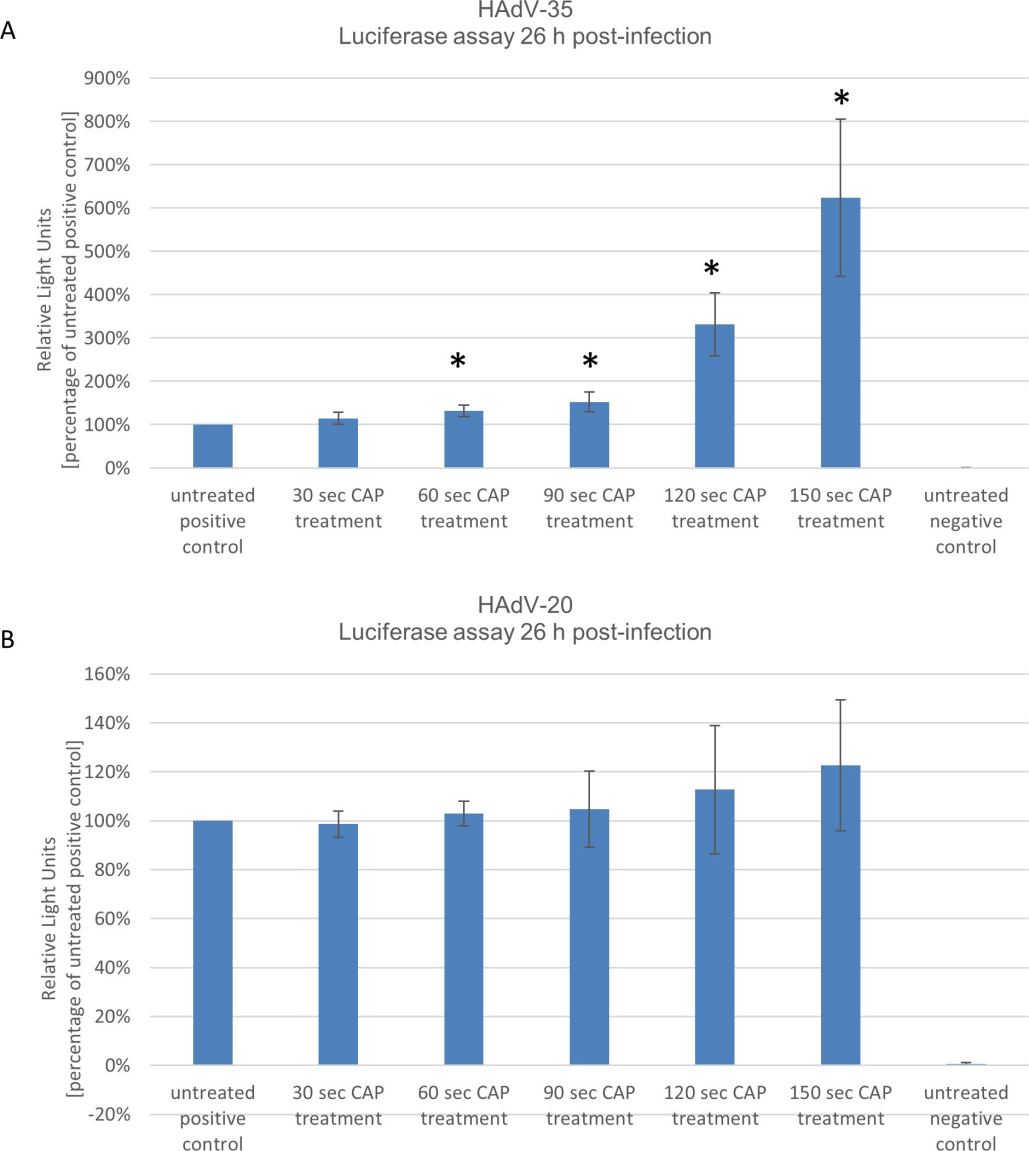
Human adenovirus (HAdV) types HAdV-35 and HAdV-20 show increased transduction efficiencies after cold atmospheric plasma (CAP) treatment. 26 hrs after transduction with CAP treated virus luciferase values were determined. Positive control: untreated virus; negative control: no virus. Mean +/- standard deviation are shown. * P-value of <0.05 was considered to be significant. Experiments were performed in triplicates and repeated 3 times.

As shown in **Fig 3A** and **Fig 3B** plasma treatment had no influence on adenovirus driven luciferase expression for HAdV-4 and HAdV-50 at any time point after CAP treatment. Even after 120 and 150 seconds of CAP treatment no significant difference in transduction efficiencies was observed. In sharp contrast, CAP treatment had a positive effect on luciferase expression for HAdV-35 and HAdV-20. As shown in **Fig 4A** after 150 seconds luciferase levels increased up to 8-fold compared to the positive control. We observed a slight increase of transduction efficiencies for HAdV-20 after CAP treatment as depicted in **Fig 4B**. Overall, these results suggest that CAP has a type dependent effect on adenoviruses and that transduction efficiencies of adenoviruses can be even increased after CAP treatment.

**Fig 5:**
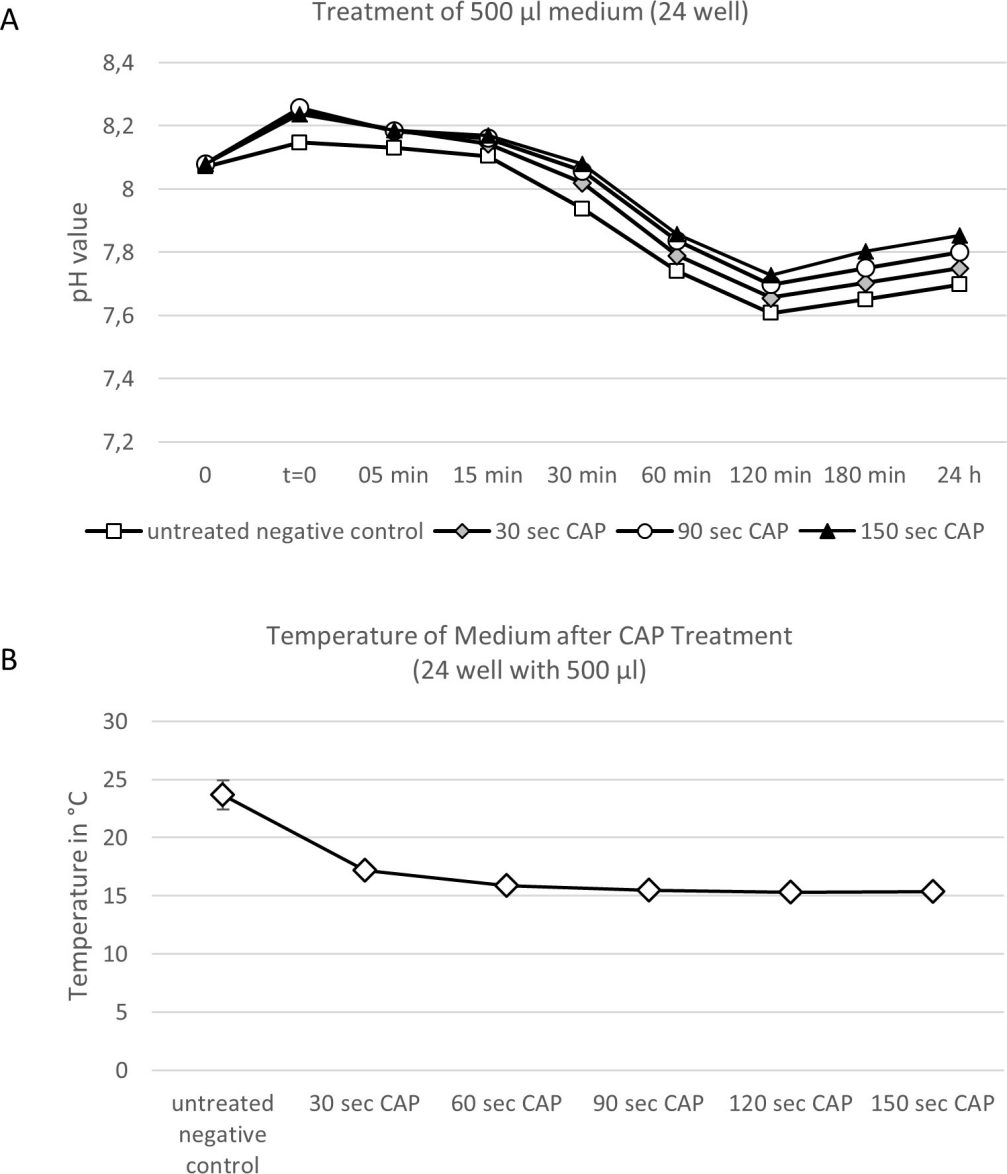
Treatment with cold atmospheric influences pH and temperature of aqueous solutions used in this study. **(A)** pH values after CAP treatment. 500 μl medium were treated by different time doses (no treatment, 30, 60, 90, 120, and 150 seconds) without adding virus. A distance of 15 mm to the medium surface was maintained and pH values were measured 5, 15, 30, 60, 120, 180 and 24 hours after treatment. **(B)** Temperature measurement after CAP treatment. A digital temperature measurement was performed 30, 60, 90, 120 and 150 after CAP treatment. Experiments were performed in triplicates and repeated 3 times. Mean +/- standard deviation are shown.

To get an idea about the mechanism related to the effect of CAP on the virus or the aqueous solution the virus is contained we performed two experiments analyzing the effect of CAP on the pH value and the temperature of the treated medium. We found that the pH values of CAP treated samples were slightly increased for all samples. For instance there was an increase in pH values of up to 0.15 (from pH 7.70 to pH 7.85) for samples treated for 150 seconds with CAP after 180 minutes and 24 hours (**Fig 5A**). In addition we measured temperatures of CAP treated solutions. We observed that the temperature of the aqueous solutions decreases to up to 10 degrees after CAP treatment (**Fig 5B**).

## Discussion

The application of therapeutic cold or nonthermal plasmas requires that they operate at atmospheric pressure. Three types of plasma sources are possible under these conditions, namely barrier discharges, corona discharges and plasma jets [12]. The kINPen med (neoplas GmbH, Greifswald, Germany) which has been used in this study belongs to the plasma jets. Weltmann and colleagues [12] described the plasma jets as a gas nozzle applied with one or two electrodes. Here, the plasma is generated inside the nozzle and transported to the object to be treated by a gas flow. Besides the different types of plasma engines the mainly differ is in electrode configuration, type of gas, and frequency of applied voltage. If different results from other studies are compared, the manufacturing process of the respective CAP must be taken into consideration.

In the present study we found that antiviral effects on different HAdVs are type-dependent. Another study explored the effect of barrier discharge plasma solely on HAdV-5 [13] and in concordance to our study is was found that treatment with CAP resulted in a dose-dependent reduction in viral load. However, in addition to this previous study we found that other human adenovirus types react differently with respect to transduction efficiencies after CAP treatment. Considering the fact that adenoviral capsids as non-enveloped viruses are relatively stable to moderate heat, UV light and pH changes [14, 15] it can only be speculated what the reasons for the observed phenomenon are. As shown in **Fig 5** CAP treatment of the medium slightly increased its pH values (**Fig 5A**) and we observed that the temperature of the aqueous solutions decreased to up to 10 degrees after CAP treatment (**Fig 5B**). However, we believe that a slight increase in pH values and decrease in temperature has no dramatic effect on stability of adenoviruses. The observed change in pH value and temperature in our study most likely has no effect on physical stability of adenoviruses which was also shown in a previous study for HAdV-5[14]. However, further studies need to be performed to experimentally prove this.

Potentially slightly differing surface proteins of different adenovirus types may react differently after CAP treatment leading to different protein structure changes. This may result in changed transduction efficiencies at different steps of virus uptake.

There is only a limited amount of studies on inactivation of viruses by CAP and effects seem to be dependent on different factors. For instance, when analyzing the effect of the argon gas plasma jet on norovirus a dose-dependent antiviral effect was found which can be regulated by the time or distance of the plasma jet to the fluid to be treated [5]. A study by Aboubakr and colleagues [16] referring to noroviruses described that smaller antiviral effects on norovirus can be achieved by pH reduction or H_2_O_2_, O_3_ and NO_2_ treatment [5, 16]. In addition the authors presented an alternative mechanism because it was exemplified for the amino acid histidine that it can be oxidized after CAP treatment, which may have an effect on virus infectivity. Therefore, future studies should analyze whether different adenovirus types also react differently on factors related to CAP treatment.

Here we used non-human Chinese ovarian hamster (CHO) cells to investigate the effect of CAP on different adenovirus types. The rational for choosing this non-human cell line was the fact that it is historically and routinely used to study uptake of adenoviruses and that human adenoviruses are replication-deficient in this cell line. This feature makes CHO cells a perfect model to study uptake of adenoviruses which then correlates with adenovirus derived transgene expression (here luciferase). A cell system which is permissive for adenovirus infection would make it difficult to analyze virus transduction efficiencies 26 hours post-infection, because massive replication of adenovirus genomes (up to 10.000 genomes per cell) and spread of virions take place and this would result in saturated luciferase levels. Here we exclusively explored luciferase levels expressed from the transduced virus genomes and correlated this with the viral load within the cells. Although there are also other methods to measure uptake of adenoviruses such as quantitative real-time PCR, our historical data [17] and our experiences demonstrated that measurement of luciferase values represents a robust technique to measure virus uptake.

Our findings may have important implications for antiviral therapies and approaches in molecular medicine. In the latter case in may be interesting to increase virus transduction efficiencies in hard-to-transduce cells. For instance increasing transduction efficiencies of HAdV on hematopoietic stem cells may have important consequences for gene therapeutic approaches. With respect to antiviral agents in medical applications CAP accessible sites could be treated and here we provide examples of diseases in the oral cavity. For instance CAP may be applied to treat the most common inflammation in the oral cavity such as such as infections herpes simplex virus type 1 (HSV-1) and Epstein-Barr-Virus (EBV) which are associated with severe acute periodontal inflammations [18]. Furthermore frequent reactivations with HSV-1 occur resulting in the manifestation visible as herpes labialis and oral manifestations of childhood viral diseases such as hand-foot-and-mouth disease caused by coxsackievirus or chickenpox induced by varicella zoster virus are possible viral target diseases for CAP treatment. Another target disease may be presented by aphthous ulcerations which are painful lesions of the oral cavity. Although the cause and etiology is largely unexplained and multifactorial, there are hints that aphthous ulcerations, especially herpetiform aphtous ulcerations may be attributed to viral infections. Coxsackievirus, HIV, HHV-8, CMV, EBV, HPV HSV-1 [19] and also adenoviruses are also suspected to cause herpetiform aphthae [20, 21].

In summary we observed an adenovirus type dependent effect of CAP differences and further studies need to investigate this phenomenon mechanistically. Potential capsid proteins oxidized by CAP may be the key to understanding the differential response of adenoviruses subtypes. These topics should be investigated in further studies.
